# *Codium fragile* Suppressed Chronic PM_2.5_-Exposed Pulmonary Dysfunction via TLR/TGF-β Pathway in BALB/c Mice

**DOI:** 10.3390/antiox12091743

**Published:** 2023-09-10

**Authors:** Tae Yoon Kim, Jong Min Kim, Hyo Lim Lee, Min Ji Go, Seung Gyum Joo, Ju Hui Kim, Han Su Lee, Won Min Jeong, Dong Yeol Lee, Hyun-Jin Kim, Ho Jin Heo

**Affiliations:** 1 Division of Applied Life Science (BK21), Institute of Agriculture and Life Science, Gyeonsang National University, Jinju 52828, Republic of Korea; kty8747@naver.com (T.Y.K.); myrock201@gnu.ac.kr (J.M.K.); gyfla059@gnu.ac.kr (H.L.L.); alswl9245@gnu.ac.kr (M.J.G.); s716g@naver.com (S.G.J.); zkfkapflove@nate.com (J.H.K.); ns3005@naver.com (H.S.L.); hyunjkim@gnu.ac.kr (H.-J.K.); 2 Research & Development Team, Gyeongnam Anti-Aging Research Institute, Sancheong 52215, Republic of Korea; jwm5618@gari.or.kr (W.M.J.); dylee1984@gari.or.kr (D.Y.L.)

**Keywords:** *Codium fragile*, particulate matter, pulmonary inflammation fibrosis, toll-like receptor

## Abstract

This study investigated the ameliorating effect of the aqueous extract of *Codium fragile* on PM_2.5_-induced pulmonary dysfunction. The major compounds of *Codium fragile* were identified as palmitic acid, stearic acid, and oleamide using GC/MS^2^ and hexadecanamide, oleamide, and 13-docosenamide using UPLC-Q-TOF/MS^E^. *Codium fragile* improved pulmonary antioxidant system deficit by regulating SOD activities and reducing GSH levels and MDA contents. It suppressed pulmonary mitochondrial dysfunction by regulating ROS contents and mitochondrial membrane potential levels. It regulated the inflammatory protein levels of TLR4, MyD88, p-JNK, p-NF-κB, iNOS, Caspase-1, TNF-α, and IL-1β. In addition, it improved the apoptotic protein expression of BCl-2, BAX, and Caspase-3 and attenuated the fibrous protein expression of TGF-β1, p-Smad-2, p-Smad-3, MMP-1, and MMP-2. In conclusion, this study suggests that *Codium fragile* might be a potential material for functional food or pharmaceuticals to improve lung damage by regulating oxidative stress inflammation, cytotoxicity, and fibrosis via the TLR/TGF-β1 signaling pathway.

## 1. Introduction

Fine dust contains several substances, including organic carbon, carbonaceous aerosols, metals and metalloids (MMs), and inorganic ions, that can negatively affect respiratory health [1]. In particular, fine dust particles, a major component of particulate matter (PM), are inhaled into the respiratory tract and interact with lung tissue [2]. These interactions promote the production of transforming growth factor-β1 (TGF-β1), a tissue growth factor, causing inflammatory responses and fibrotic processes [3]. TGF-β1 secreted from pulmonary cells affects surrounding tissues, and these effects are related to inflammation and fibrosis caused by PM [4]. When exposure to fine dust occurs, fibroblasts in lung tissues abnormally increase, and excessive accumulation of fibrous proteins such as collagen causes excessive fibrosis of tissues [5]. Lung tissue damage caused by fine dust continuously activates TGF-β1, which promotes fibroblast differentiation and collagen synthesis, thereby promoting fibrosis [6]. When PM_2.5_, which classified as a size smaller than 2.5 μm, particles are inhaled into the respiratory tract, certain cells in lung tissue, such as lung epithelial and immune cells, interact with the PM_2.5_ particles [5]. PM_2.5_ particles adsorb various elements on their surface, and these elements activate Toll-like receptors (TLR), which play a major role in the host’s immune system [1]. Activated TLRs initiate signal transduction pathways inside cells, thereby producing various inflammatory response-inducing factors [7]. Activation of the TLR pathway in this process causes continuous secretion of TGF-β1, and the interaction between PM_2.5_ and TLRs leads to pulmonary fibrosis and inflammatory responses such as nuclear factor kappa-light-chain-enhancer of the activated B cell (NF-κB) pathway [8]. This inflammatory response induces oxidative stress and the production of proinflammatory mediators, leading to various diseases such as asthma, chronic obstructive pulmonary disease (COPD), and cancer [6]. To reduce damage to lung tissue from PM_2.5_, it is most important to avoid direct exposure [4]. However, since PM inhalation cannot be completely inhibited, it is important to reduce the toxicity of PM in advance with the consumption of or treatment with natural materials [9].

*Codium fragile*, a marine green alga belonging to the family *Codiaceae*, is cultivated and used for food in various countries such as the Republic of Korea, China, and Japan, and in North America [10]. *Codium fragile* contains various bioactive phenolic compounds, such as salicylic acid, p-coumaric acid, tamarixetin, morin, naringenin, kaempferol, and quercetin, and polysaccharides such as sulfated galactan [11]. It also has been reported that *Codium fragile* shows bioactive activities such as antioxidant activity, anti-diabetes, osteoarthritis inhibitory effect, hepato-protective effect, and anti-inflammatory effect [12,13]. However, there are few studies related to the protective effect of *Codium fragile* on PM_2.5_-induced lung disease. In a previous study, the aqueous extract of *Codium fragile* significantly protected against PM_2.5_-induced cytotoxicity in pulmonary A549 cells and nasal RPMI2650 cells [14]. Thus, this study was conducted to estimate the ameliorating effect of the aqueous extract of *Codium fragile* against PM_2.5_-induced pulmonary damage.

## 2. Materials and Methods

### 2.1. Sample Preparation

*Codium fragile* used in this study was obtained from Yeosu (Republic of Korea) in February 2018. The samples were washed to remove salt and impurities until the salt concentration reached 0%. The desalted samples were lyophilized using a vacuum drier (Operon, Gimpo, Republic of Korea) and extracted with 50-fold distilled water at 40 °C. The extracted samples were filtered with a No. 2 filter (Whatman Inc., Kent, UK) and concentrated using an evaporator with a vacuum (N-N series, Eyela Co., Tokyo, Japan). The re-lyophilized samples were stored at −20 °C until used.

### 2.2. Gas Chromatography–Tandem Mass Spectrometry (GC/MS^2^)

The dried aqueous extract of *Codium fragile* was extracted with 80% methanol using a bullet blender (Next Advance Inc., Averill Park, NY, USA) and sonicated for 20 min. The extracted sample was reacted with methoxyamin at 37 °C for 90 min, and the mixture was incubated with BSTFA 70 °C for 30 min. The mixture was centrifuged at 14,000× *g* for 10 min, and the supernatant filtered by a 0.45 µm membrane filter was used for identification. The contents of physiological compounds were identified using GC/MS^2^ (GC, Agilent 7890A; MS^2^, Agilent 5975C, Agilent, Santa Clara, CA, USA) on a capillary column (30 m × 250 µm, 0.25 µm, DB-5MS DB-5MS Inert Column, Agilent). Initially, samples (1.0 μL) were injected in splitless mode (50:1). The injection temperature was preserved at 260 °C, and the protocol was conducted with helium gas (1.0 mL/min). Using a temperature control system, the initial column oven temperature was set at 40 °C for 5 min, then 10 °C/min to 120 °C for 8 min, then 60 °C/min to 300 °C for 3 min, and then 300 °C for 0.5 min (total run time: 16.5 min). The MS conditions used were as follows: acquisition mode, scan (40 to 250 *m*/*z*); quadrupole temperature, 150 °C. The GC/MS^2^ system was analyzed using library software (NIST MS search 2.2, National Institute of Standards and Technology, Gaithersburg, MD, USA).

### 2.3. Ultra-Performance Liquid Chromatography–Quadrupole Time-of-Flight Mass Spectrometry (UPLC-Q-TOF/MS^E^)

The extracted sample was fractionated using n-hexane, chloroform, and ethyl acetate to remove the impurities, and a fraction from distilled water of *Codium fragile*, which contained the highest content of oleamide, was used for identification. The dried sample of fraction from distilled water dissolved in methanol filtered through a 0.45 µm membrane filter and analyzed using UPLC-Q-TOF/MS^E^ (Vion, Waters Corp., Milford, MA, USA) with a BEH C_18_ column (100 × 2.1 mm, 1.7 µm; Waters Corp.). The mobile phases consisted of solvent A (0.1% formic acid in distilled water) and solvent B (0.1% formic acid in acetonitrile) during analysis, and gradient conditions were as follows: 1% B at 0–1 min; 1–100% B at 1–8 min; 100% B at 8–9 min; 100–1% B at 9–9.5 min; and 1% B at 9.5–12 min. 

### 2.4. High-Performance Liquid Chromatography (HPLC)

The dried sample dissolved in methanol was filtered through a 0.45 µm membrane filter and analyzed using HPLC (Ultimate 3000 series, Dionex, Sunnyvale, CA, USA). HPLC separation of oleamide was conducted using a C_18_ column (250 × 4.6 mm, 5.0 µm, YMC-Triart, YMC, Kyoto, Japan) with a flow rate of 1.0 mL/min. The mobile phases consisted of solvent A (distilled water) and solvent B (acetonitrile), and the analysis conditions were as follows: a gradient elution of 50% A and 50% B at 0–0.1 min; 50–0% A and 50–100% B at 0.1–20 min; and 0% A and 100% B at 20–30 min. The injected volume was 20 μL, and the wavelength of the UV detector was analyzed using a diode array detector at 203 nm at 40 °C to measure the oleamide. The detected wavelength was compared to a standard compound to determine similarity.

### 2.5. Animal Experimental Design

**Figure 1:**
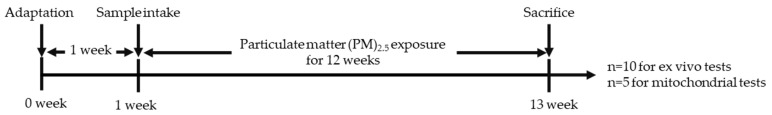
Experimental design of the particulate matter (PM)_2.5_ exposure model and ex vivo tests for PM_2.5_-induced mice.

BALB/c mice (6 week olds, male) were obtained from Samtako (Osan, Republic of Korea). The animals were divided into 3 or 4 per cage and controlled in standard laboratory conditions (12 h light/dark cycle; 55% humidity; 22 ± 2 °C). Experimental groups were divided into 6 groups (n = 10; 5 for ex vivo tests; 5 for mitochondrial tests) as a sham control (sham) group (chamber exposure-, sample intake-), normal control (NC) group (a clean air-exposure+, sample intake-), normal sample (NS) group (a clean air-exposure+, sample intake+; 40 mg/kg of body weight), PM group (PM_2.5_ air-exposure+, sample intake-), and the *Codium fragile* groups (PM_2.5_ air-exposure+, sample intake+; 50 and 100 mg/kg of body weight; CF50 and CF100, respectively). The *Codium fragile* was dissolved in pure water and orally fed using a stomach tube once a day for 12 weeks. PM_2.5_ (mean diameter: 1.06 µm) was obtained from Power Technology INC. (Arizona Test Dust, Arden Hills, MN, USA). The animal was exposed to PM_2.5_ (500 μg/m^3^) in the chamber for 5 h/day for 12 weeks. All procedures were conducted in accordance with the Institutional Animal Care and Use Committee of Gyeongsang National University (certificate: GNU-210803-M0069, approved on 3 August 2021) and the Policy of the Ethical Committee of Ministry of Health and Welfare, Republic of Korea. The experimental design was presented as follows (Figure 1).

### 2.6. Antioxidant System Activity

#### 2.6.1. Preparation of Lung Tissues

After all mice were fasted for 12 h, experimental animals were sacrificed by CO_2_ inhalation. Lung tissues were immediately isolated for ex vivo tests, and tissues were homogenized with 10 times the volume of phosphate-buffered saline (PBS, pH 7.4) or phosphate buffer (pH 7.8). The protein concentration of the obtained sample was evaluated according to the previous study [15].

#### 2.6.2. Superoxide Dismutase (SOD) Activity

To assess the SOD activities, the lung tissues homogenized in PBS buffer were centrifuged at 400× *g* for 10 min at 4 °C, and the obtained pellet was extracted using 1 × cell extraction buffer with 20% (*v*/*v*) Triton X-100 and 200 mM phenylmethylsulfonyl fluoride. The mixtures incubated for 30 min on ice were centrifuged at 10,000× *g* for 10 min at 4 °C. The obtained supernatants were used for SOD activities using a SOD commercial kit (Sigma-Aldrich Chemical Co., St. Louis, MO, USA).

#### 2.6.3. Reduced Glutathione (GSH) Contents

To assess the reduced GSH contents, the lung tissues homogenized in phosphate buffer (pH 7.8) were mixed with 200 μM phosphate buffer (pH 6–7) and centrifuged at 10,000× *g* for 15 min at 4 °C. The protein concentration of the supernatant was quantified according to the Bradford method [15]. Then, the supernatants and 5% metaphosphoric acid were mixed and centrifuged at 2000× *g* for 2 min at 4 °C. The supernatant was incubated with 0.26 M Tris-HCl (pH 7.5), 0.65 N NaOH, and 1 mg/mL o-phthaldialdehyde. The fluorescence of reactants was measured at 360 nm (excitation wavelength) and 430 nm (emission wavelength) using a fluorometer (Infinite F200, Tecan, Mannedorf, Switzerland) [16].

#### 2.6.4. Malondialdehyde (MDA) Contents

To assess the MDA contents, the lung tissues homogenized in PBS buffer were centrifuged at 2350× *g* for 10 min at 4 °C. The supernatants were mixed with 1% phosphoric acid and 0.67% thiobarbituric acid, and these mixtures were incubated at 95 °C for 1 h. After that, the MDA contents were measured at 532 nm using a microplate reader (Epoch2, BioTek, Winooski, VT, USA) [16].

### 2.7. Mitochondrial Function Activity

#### 2.7.1. Isolation of Mitochondria

Lung tissues were homogenized in mitochondrial isolation buffer (215 mM mannitol, 75 mM sucrose, 0.1% bovine serum albumin, and 20 mM HEPES sodium salt (pH 7.2) and 1 mM EGTA). The homogenized tissues were centrifuged at 1300× *g* for 5 min at 4 °C to obtain the supernatant. The supernatants were re-centrifuged at 13,000× *g* for 10 min at 4 °C, and the pellets were mixed with 0.1% digitonin and mitochondrial isolation buffer containing 1 mM EGTA for 5 min. The mixtures were centrifuged at 13,000× *g*, for 15 min at 4 °C. The re-obtained pellets mixed with mitochondrial isolation buffer were used for the evaluation of mitochondrial functions.

#### 2.7.2. Mitochondrial Reactive Oxygen Species (ROS) Content

To assess the mitochondrial ROS levels, mitochondrial extracts were reacted to a respiration buffer containing 125 mM KCl, 2 mM KH_2_SO_4_, 2.5 mM malate, 20 mM HEPES, 1 mM MgCl_2_, 5 mM pyruvate, 500 μM EGTA, and 25 μM DCF-DA. These reactants were incubated in a dark room for 20 min, and fluorescence was measured at 485 nm (excitation wavelength) and 535 nm (emission wavelength) [17].

#### 2.7.3. Mitochondrial Membrane Potential (MMP) Levels

To assess the MMP level, mitochondrial extracts were reacted to a mitochondrial isolation buffer with 5 mM pyruvate, 5 mM malate, and 1 μM JC-1. The reactants were incubated in a dark room for 20 min, and fluorescence was measured at 530 nm (excitation wavelength) and 590 nm (emission wavelength) [17].

### 2.8. Western Blot

Lung tissues were homogenized using ProtinEx™ Animal cell/tissue (Gene All Biotechnology, Seoul, Republic of Korea) with 1% protease inhibitor and phosphatase inhibitor. The homogenized tissues were separated by sodium dodecyl sulfate–polyacrylamide gel electrophoresis (SDS-PAGE) and transferred to a PVDF membrane (Millipore, Billerica, MA, USA). The transferred membranes were treated with 5% skim milk for 1 h and washed using 1 × tris-buffer saline with 0.1% Tween^®^ 20 (TBST) buffer 3 times for 10 min. The blocked membranes were incubated in a primary antibody solution for 12 h at 4 °C. The incubated membranes were reacted with a secondary antibody (1:2500) for 1 h. The chemiluminescence of each protein was detected using an image analyzer (iBright™ CL1500 instrument, Invitrogen, Carlsbad, CA, USA). Antibody information is presented in Table 1.

### 2.9. Statistical Analysis

All experimental results were expressed as mean ± standard deviation (SD) and assessed by one-way analysis of variance (ANOVA) to analyze significant differences. Each set of data was evaluated using Duncan’s new multiple range test (*p* < 0.05) with the statistical program (SAS version 9.4, SAS Institute Inc., Cary, NC, USA). Significant differences were statistically presented as different small letters.

## 3. Results

### 3.1. Physiological Compounds in Codium fragile

**Figure 2:**
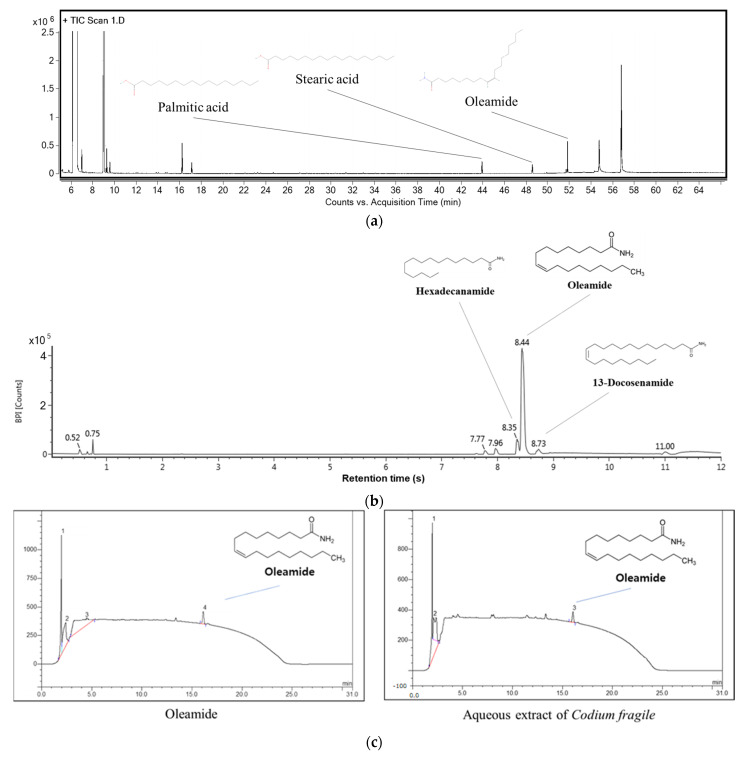
UPLC Q–TOF/MS^E^ chromatography in negative ion mode of *Codium fragile*. (**a**) MS^2^ spectra of gas chromatography (GC)/MS^2^; (**b**) Ultra-Performance Liquid Chromatography–Quadrupole Time-of-Flight Mass Spectrometry (UPLC-Q-TOF/MS^E^); (**c**) High-Performance Liquid Chromatography (HPLC).

The compounds of the aqueous extract of *Codium fragile* were qualitatively identified using GC/MS^2^ (Figure 2a and Table 2) and UPLC-Q-TOF/MS^E^ (Figure 2b and Table 3) and quantitatively identified using HPLC (Figure 2c). The MS^2^ spectra of GC/MS^2^ were analyzed as compound 1: 328 *m*/*z* (retention time (RT): 43.92 min); compound 2: 356 *m*/*z* (RT: 48.58 min); and compound 3: 330 *m*/*z* (RT: 51.82 min). These compounds were tentatively identified as palmitic acid (PubChem CID:985, compound 1), stearic acid (PubChem CID:5281, compound 2), and oleamide (PubChem CID: 5283387, compound 3). The MS^2^ spectra of UPLC-Q-TOF/MS^E^ were analyzed as compound 1: 256 *m*/*z* (RT: 8.35 min); compound 2: 282 *m*/*z* (RT: 8.44 min); and compound 3: 338 *m*/*z* (RT: 8.73 min). These detected compounds were tentatively identified as hexadecanamide (PubChem CID: 69,421, compound 1), oleamide (PubChem CID: 5283387, compound 2), and 13-docosenamide (PubChem CID: 5,365,371, compound 3). In the result of HPLC, oleamide contents were 7.74 mg/g dried weight compared to the retention time and UV-VIS spectrum of a standard material.

### 3.2. Effect of Codium fragile on Antioxidant System Biomarkers

**Figure 3:**
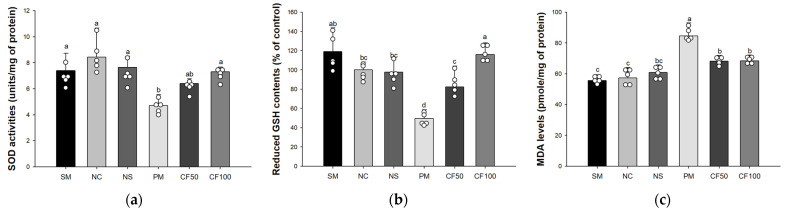
Protective effect of the aqueous extract of *Codium fragile* on PM_2.5_-induced biochemical changes related to antioxidant system. (**a**) Superoxide dismutase (SOD) contents; (**b**) reduced glutathione (GSH) level; (**c**) malondialdehyde (MDA) contents. The results shown are mean ± SD (n = 5). Data were statistically considered at *p* < 0.05, and different small letters represent statistical differences.

As a result of analyzing SOD activities, there were no significant differences between the sham group (7.39 unit/mg of protein), NC group (8.44 unit/mg of protein), and NS group (7.65 unit/mg of protein) (Figure 3a). The SOD activities of the PM group (4.71 unit/mg of protein) significantly decreased compared to the NC groups. However, the CF50 and CF100 groups (6.40 unit/mg of protein and 7.31 unit/mg of protein, respectively) were significantly ameliorated compared to the PM group. As a result of analyzing reduced GSH levels, there were no significant differences between the sham group (119.04% of control), the NC group (100% of control), and the NS group (97.83% of control) (Figure 3b). The GSH contents of the PM group (49.59% of control) were significantly decreased compared to the NC group. However, the CF50 and CF100 groups (82.24% of control and 115.99% of control, respectively) were significantly ameliorated compared to the PM group. As a result of analyzing MDA contents, there were no significant differences between the sham group (55.70 pmole/mg of protein), the NC group (57.30 pmole/mg of protein), and the NS group (60.90 pmole/mg of protein) (Figure 3c). The MDA levels of the PM group (84.60 pmole/mg of protein) were significantly increased compared to the NC group. However, the CF50 and CF100 groups (68.30 pmole/mg of protein and 68.50 pmole/mg of protein, respectively) were significantly ameliorated compared to the PM group.

### 3.3. Effect of Codium fragile on Mitochondrial Activity

**Figure 4:**
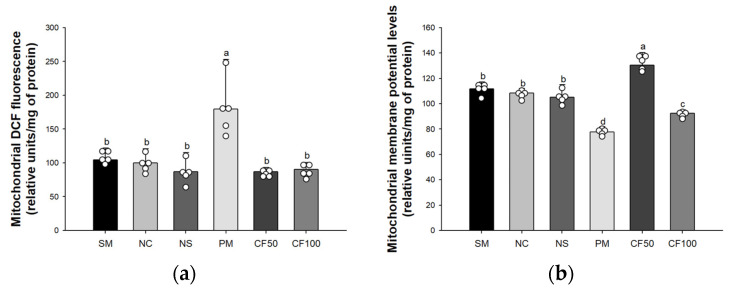
Protective effect of the aqueous extract of *Codium fragile* on PM_2.5_-induced mitochondrial dysfunction. (**a**) Reactive oxygen species (ROS) contents; (**b**) mitochondrial membrane potential (MMP) levels. The results shown are mean ± SD (n = 5). Data were statistically considered at *p* < 0.05, and different small letters represent statistical differences by one-way analysis of variance (ANOVA) and Duncan’s new multiple range test.

As a result of analyzing mitochondrial ROS levels, there were no significant differences between the sham group (104.31% of control), the NC group (100% of control), and the NS group (87.06% of control). The ROS levels of the PM group (179.70% of control) were significantly increased compared to the NC group (Figure 4a). However, the CF50 and CF100 groups (87.19% of control and 90.52% of control, respectively) were significantly ameliorated compared to the PM group. As a result of analyzing MMP levels, there were no significant differences between the Sham group (103.20% of control), the NC group (100% of control), and the NS group (96.96% of control). The MMP levels of the PM group (71.70%) were significantly decreased compared to the NC group (Figure 4b). However, the CF50 and CF100 groups (120.35% and 85.40%, respectively) were significantly ameliorated compared to the PM group.

### 3.4. Effect of Codium fragile on PM_2.5_-Induced Pulmonary Inflammatory-Related Factors

**Figure 5:**
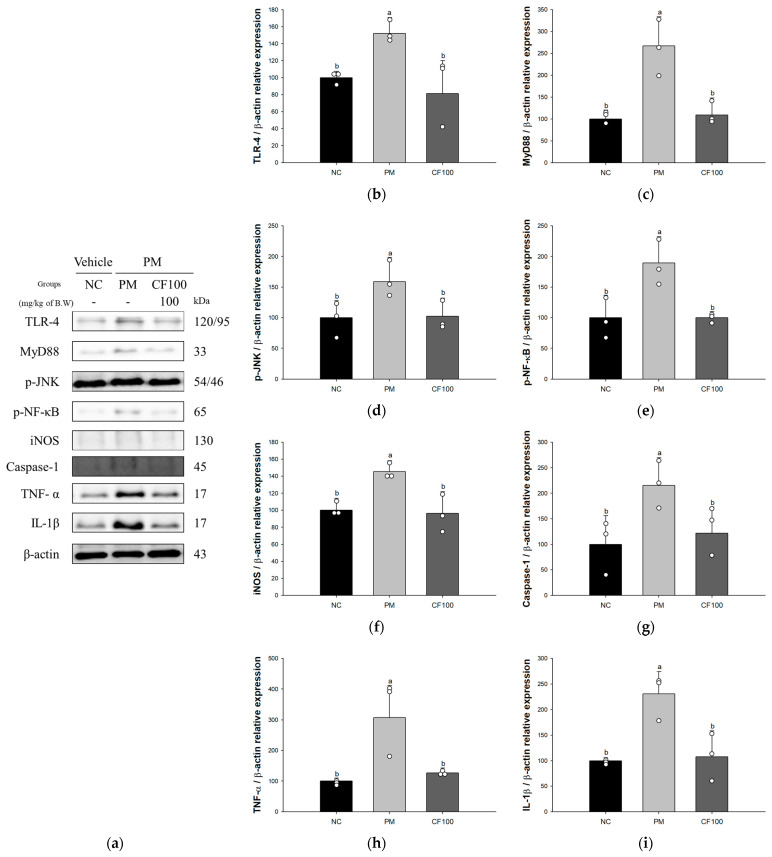
Regulatory effect of the aqueous extract of *Codium fragile* on PM_2.5_-induced inflammatory related protein expression in Western blot. (**a**) Western blot band image; protein expression levels of (**b**) Toll-like receptors 4 (TLR4); (**c**) myeloid differentiation primary response 88 (MyD88); (**d**) phosphorylated c-Jun N-terminal kinase (p-JNK); (**e**) phosphorylated nuclear factor kappa-light-chain-enhancer of the activated B cell (p-NF-κB); (**f**) inducible nitric oxide synthase (iNOS); (**g**) Caspase-1; (**h**) tumor necrosis factor-α (TNF-α); (**i**) interleukin-1β (IL-1β). The results shown are mean ± SD (n = 3). Data were statistically considered at *p* < 0.05, and different small letters represent statistical differences by one-way analysis of variance (ANOVA) and Duncan’s new multiple range test.

The expression levels of TLR4 (152.14%), myeloid differentiation primary response 88 (MyD88) (266.74%), phosphorylated c-Jun N-terminal kinase (p-JNK) (158.73%), p-NF-κB (189.59%), inducible nitric oxide synthase (iNOS) (145.44%), Caspase-1 (215.34%), tumor necrosis factor-α (TNF-α) (307.61%), and interleukin-1β (IL-1β) (230.52%) of the PM group were significantly increased compared to NC group (Figure 5). However, the CF100 group (81.19%, 109.14%, 102.43%, 100.17%, 96.45%, 121.76%, 126.83%, and 107.73%, respectively) was ameliorated compared to the NC group.

### 3.5. Effect of Codium fragile on PM_2.5_-Induced Pulmonary Apoptosis-Related Factors

**Figure 6:**
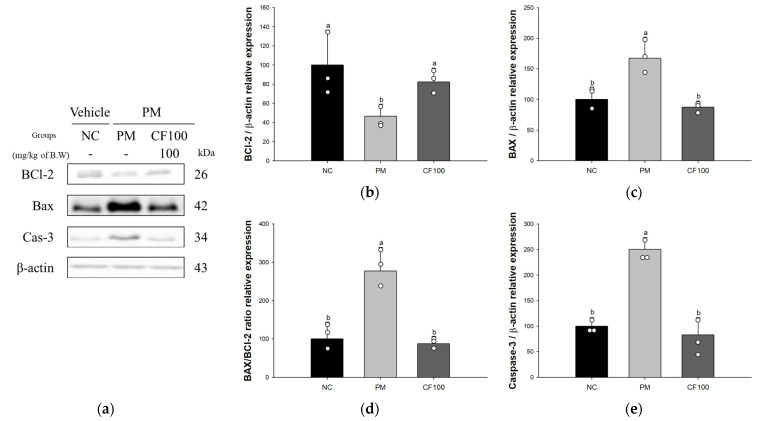
Regulatory effect of the aqueous extract of *Codium fragile* on PM_2.5_-induced apoptosis-related protein expression in Western blot. (**a**) Western blot band image; protein expression levels of (**b**) B-cell lymphoma 2 (BCl-2); (**c**) BCl-2 associated X (BAX); (**d**) BAX/BCl-2 ratio; (**e**) Caspase-3. The results shown are mean ± SD (n = 3). Data were statistically considered at *p* < 0.05, and different small letters represent statistical differences by one-way analysis of variance (ANOVA) and Duncan’s new multiple range test.

The expression level of B-cell lymphoma 2 (BCl-2) (46.53%) of the PM group was significantly decreased compared to the NC group (Figure 6). However, the CF100 group (82.50%) was ameliorated compared to the NC group. The expression levels of BCl-2 associated X (BAX) (167.78%), BAX/BCl-2 ratio (276.85%), and Caspase-3 (250.18%) of the PM group were significantly increased compared to the NC group. However, the CF100 group (87.52%, 87.44%, and 82.81%, respectively) was ameliorated compared to the NC group.

### 3.6. Effect of Codium fragile on PM_2.5_-Induced Pulmonary Fibrosis-Related Factors

**Figure 7:**
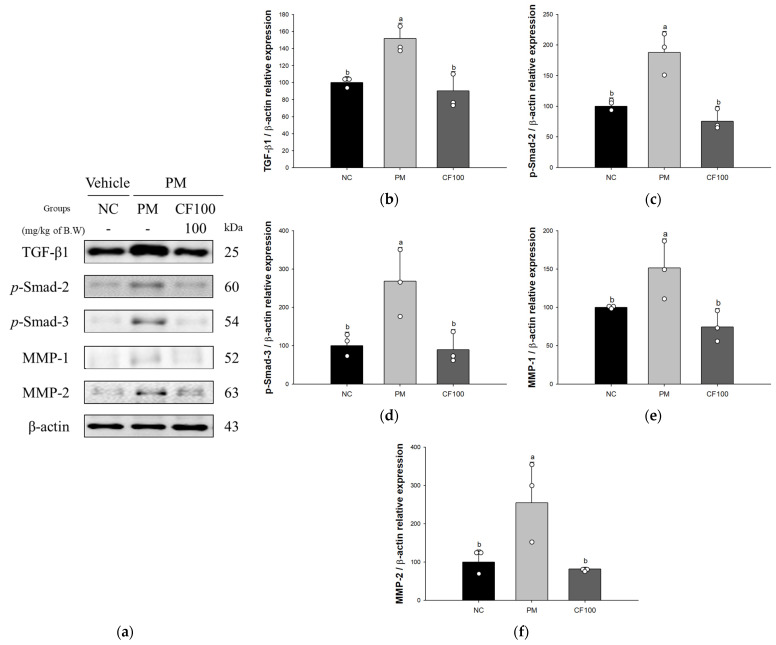
Regulatory effect of the aqueous extract of *Codium fragile* on PM_2.5_-induced pulmonary fibrosis related protein expression in Western blot. (**a**) Western blot band image; protein expression levels of (**b**) transforming growth factor-β1 (TGF-β1); (**c**) phosphorylated small mothers against decapentaplegic (p-Smsd)-2; (**d**) p-Smad-3; (**e**) matrix metalloproteinase-1 (MMP-1); (**f**) matrix metalloproteinase-2 (MMP-2). The results shown are mean ± SD (n = 3). Data were statistically considered at *p* < 0.05, and different small letters represent statistical differences by one-way analysis of variance (ANOVA) and Duncan’s new multiple range test.

The expression levels of TGF-β1 (151.86%), phosphorylated small mothers against decapentaplegic (p-Smad)-2 (187.56%), p-Smad-3 (268.35%), matrix metalloproteinase-1 (MMP-1) (151.59%), and matrix metalloproteinase-2 (MMP-2) (254.89%) of the PM group were significantly increased compared to the NC group (Figure 7). However, the CF100 group (90.44%, 75.66%, 89.33%, 74.45%, and 81.74%, respectively) was ameliorated compared to the NC group.

## 4. Discussion

Environmental air pollutant PM_2.5_ causes various health problems including respiratory disease, asthma, COPD, and lung cancer [6]. Chronic exposure to PM_2.5_ causes continuous inflammation, loss of oxidative stress scavenging function, and pulmonary fibrosis, which ultimately cause death of lung tissue and various lung diseases [4]. In particular, depending on MMS and other compositions, fine dust exhibits various toxicities and easily damages lung tissue [1]. Therefore, it is most important to block fine dust from the air, but since this is not easy, it is important to increase antioxidant activity in the body. In addition, since there are few treatments for pulmonary fibrosis, it is important to prevent it in advance through the consumption of antioxidants or natural products with strong physiological activity [9]. On the other hand, *Codium fragile* has a considerable possibility of use as an excellent natural product with various physiological activities [12]. On the other hand, studies of *Codium fragile* on fine dust toxicity related to protective effects or specific mechanisms are not clear. Thus, this study was estimated to assess the pulmonary ameliorating effect of the aqueous extract of *Codium fragile* against chronic PM_2.5_-inhalated inflammation and fibrosis in BALB/c mice.

PM_2.5_ contains various materials such as heavy metals and organic compounds that cause oxidative stress [1]. PM_2.5_, which is composed of these reactive compounds, reaches the lung tissues and causes lipid peroxidation, DNA denaturation, structural degeneration, and mitochondrial dysfunction through damage to the antioxidant system [18]. In particular, lung tissues are in direct contact with PM_2.5_ and have a structure, that is vulnerable to PM, and damage to the alveoli causes inflammatory responses by producing cytokines due to structural dysfunction. In addition, when PM_2.5_ is deposited in lung tissues, it is difficult to remove and continuously leads to damage to the antioxidant system [19]. Oxidative stress derived from PM_2.5_ increases the production of various radicals, and superoxide resulting from this process ultimately leads to the depletion of antioxidant substances such as SOD, GSH, catalase, and glutathione peroxidase (GPx) and lung damage [20]. Therefore, to estimate the protective effects of *Codium fragile* against PM_2.5_-induced oxidative stress, SOD activities, reduced GSH contents, and MDA levels were estimated in lung tissues (Figure 3). According to a previous study, *Codium fragile* contains various flavonoid compounds such as naringenin, kaempferol, catechin, and epicatechin with considerable antioxidant activities [11]. Administration of these flavonoids had significant protective effects against oxidative stress-induced cytotoxicity in the systemic antioxidant system [21]. Considerable amounts of δ-tocopherol and α-tocopherol were found in *Codium fragile,* and these tocopherols showed significant antioxidant activity against cytotoxicity [22]. Oleamide inhibited lipid peroxidation and the imbalance in the ratio of reduced/oxidized GSH in 3-nitropropionic acid-induced antioxidant damage [23]. In addition, a sulfated polysaccharide from *Codium fragile* attenuated the disruption of antioxidant enzymes such as SOD, catalase, and GPx, and inhibited cytotoxicity indicators such as aspartate aminotransferase (AST), alanine aminotransferase (ALT), and lactate dehydrogenase (LDH) in high fat diet-induced liver and kidney cytotoxicity [10,24]. Based on these results, the aqueous extract of *Codium fragile* containing various compounds with physiological activities might have an ameliorating effect against PM_2.5_-induced oxidative stress and cytotoxicity in lung tissues.

Inflammatory response and radicals generated by PM_2.5_ lead to mitochondrial dysfunction in lung tissue [25]. Repeated and chronic exposure to PM_2.5_ causes reduced mitochondrial fusion and impaired dynamics with the reduction of optic atrophy 1 (OPA1) and mitofusin2 (MFN2) [26]. PM_2.5_ causes mitochondrial morphology damage with mitochondrial swelling, vacuole formation, and crystal destruction [27]. In addition, in damaged mitochondria, PTEN-induced kinase 1 (PINK1), a mitophagy regulatory protein, accumulates in the mitochondrial outer membrane, thereby inducing an abnormal mitophagy process resulting in a decrease in mitochondrial volume, inhibition of mitochondrial respiratory function, and an increase in mitochondrial ROS levels [28]. This mitochondrial deficit induces abnormal energy metabolism in lung tissues by reducing the MMP level and damaging the sodium–potassium pump and calcium pump [26]. Thus, in this study, the protective effects of the aqueous extract of *Codium fragile* against PM_2.5_-induced mitochondrial dysfunction were confirmed, and mitochondrial ROS contents and MMP levels were ameliorated (Figure 4). Catechins, the bio-active compounds in *Codium fragile*, had a considerable protective effect against mitochondrial damage by regulating the mitochondrial complex and MMP levels [21]. Oleamide, a major compound in the aqueous extract of *Codium fragile*, regulated the inhibition of neuronal excitability with the activation of cannabinoid receptors against quinolinic acid-induced mitochondrial and synaptic dysfunction [29]. Oleamide also regulated mitochondrial dysfunction and death in 3-nitropropionic acid-induced mitochondrial deficit with the regulation of mitochondrial complex and cannabinoid receptors [23]. The administration of *Codium fragile* containing lysophosphatidyl choline and canthaxanthin promoted mitochondrial biogenesis with the regulation of peroxisome proliferator-activated receptor-gamma coactivator (PGC)-1α-related pathway [30]. Furthermore, the intake of *Codium fragile* regulated intestinal microbiota involved in mitochondrial energy metabolism such as pyruvate fermentation and glycolysis [31]. Thus, various phenolic compounds and unsaturated fatty acids in *Codium fragile* might help maintain lung health by suppressing PM_2.5_-induced mitochondrial damage related to energy metabolism.

Absorbed PM_2.5_ induces the inflammatory response in various tissues by binding into TLRs and stimulates MyD88 resulting in extensive inflammatory damage [32]. Stimulated TLRs continuously activate mitogen-activated protein kinase (MAPK), including extracellular signal-regulated kinase (ERK)1/2, p38 kinase, and JNK, and NF-κB pathway stimulating the secretion of cytokines and chemokines such as 1L-1β, interleukin-12, TNF-α, and monocyte chemoattractant protein-1. In addition, because PM_2.5_ is composed of complex components including organic carbon, radicals, carbonaceous aerosols, inorganic ions, heavy metals, and polycyclic aromatic hydrocarbons, it activates various receptors such as aryl hydrocarbon receptors, hormone receptors, angiotensin type 1 receptors as well as TLRs [32]. The activated receptors stimulate inflammatory response, hormonal imbalance, and apoptotic signal through MAPK/NF-κB/phosphoinositide 3-kinase (PI3K)/protein kinase B (Akt) pathways. These activated signals increase the gene expression of cyclooxiganse-2 (COX-2) and iNOS-producing inflammatory cytokines [32]. Therefore, to evaluate the anti-inflammatory effect of the aqueous extract of *Codium fragile*, inflammatory protein expression levels in lung tissues were confirmed, and the consumption of this extract significantly suppressed inflammation in lung tissues (Figure 5). Similar to this study, sulfated polysaccharides isolated from *Codium fragile* significantly down-regulated inflammatory indicators such as prostaglandin E2, nitric oxide, and TNF-α in RAW 264.7 cells [33]. In addition, baicalin as one of the bio-active compounds of *Codium fragile* suppressed inflammation via PI3K/sirtuin 1 (SIRT1)/MAPK/NF-κB pathway [8]. The aqueous extract from *Codium fragile* decreased nitrite production, protein expression of iNOS, matrix metalloproteinase-13, a disintegrin and metalloproteinase with thrombospondin motifs (ADAMTS)-4, and ADAMTS-5 against IL-1β-induced osteoarthritis with the regulation of the MAPK/NF-κB signal [13]. *Codium fragile* also inhibited inflammatory cytokines such as TNF-α, IL-1β, and IL-6 and nuclear translocation of NF-κB by suppressing the phosphorylation and degradation of IκB-α [34], and suppressed inflammatory indicators such as COX-2, iNOS, prostaglandin E2, and release of nitric oxide (NO) [35]. In addition, treatment of kaempferol, as one of the flavonoid compounds, significantly inhibited IgE and lipopolysaccharide-induced inflammation via nuclear factor erythroid 2-related factor 2 (Nrf2)/SHIP1 on bone marrow-derived mast cells (BMMCs) [36]. In various previous studies, *Codium fragile* might help significantly inhibit the inflammatory reaction caused by PM_2.5_, and in particular, it is judged to be able to suppress inflammation through the NF-kB pathway.

PM_2.5_ promotes the initiation of an inflammatory response as well as the generation of oxidative stress, resulting in cytotoxicity [4]. Oxidative stress induced by fine dust causes damage to the antioxidant system, dysfunction of mitochondria, and damage to lung cell membranes [16]. Damage to pulmonary cells increases the level of intracellular Ca^2+^ and results in the release of cytochrome c from inside the mitochondria [37]. This process acts as a signal for apoptosis and induces a caspase cascade by causing an imbalance of mitochondria-related proteins such as BAX, BCl-2, and BCl-Xl/BCl-2-associated death promoter homolog (Bad) [37]. Therefore, continuous and chronic exposure to PM_2.5_ stimulates intracellular apoptosis, which ultimately leads to cell death, which causes lung tissue dysfunction [38]. Therefore, to estimate the ameliorating effect of the aqueous extract of *Codium fragile*, apoptotic expression levels in lung tissues were evaluated, and the administration of *Codium fragile* significantly down-regulated pulmonary apoptosis (Figure 6). Oleamide significantly suppressed the nuclear condensation and activation of Caspase-3 in cerebellar granule neurons induced by K^+^ deprivation. However, oleamide isomers without the Δ9-cis double bond, such as elaidic acid or stearic acid, did not affect cell death [39]. In addition, rutin, one of the flavonoids of *Codium fragile*, inhibited apoptosis by regulating the expression of BCl-2/BAX ratio, Caspase-9, and cleaved poly ADP-ribose polymerase (PARP) in endometriosis development in a rat model [37]. The treatment of p-coumaric acid suppressed apoptosis signaling in ethanol-induced hepatotoxicity by attenuating the expression of BAX, caspases, AST, and LDH via the PI3K/Akt pathway [40]. In conclusion, the aqueous extract of *Codium fragile* with physiological activities significantly suppressed apoptosis and might be used as a material to protect against PM_2.5_-induced cytotoxicity. However, studies on factors related to other caspase cascades, including activated caspase-3, caspase-9, and cleaved PARP, need to be investigated in further experiments.

Fine dust in the air is absorbed into lung tissue and increases the expression of TGF-β1, which plays an important role in damage and repair signaling [4]. TGF-β1 secreted from fibroblasts and myofibroblasts activates the TGF-β1 receptor to phosphorylate Smad2/3 and stimulates the expression of MMPs [41]. Through this process, sub-signals such as type I collagen (Col1) and α-smooth muscle actin (α-SMA) related to collagen accumulation are continuously stimulated and continue to cause fibrosis and cancer [5]. In particular, heavy metals in PM_2.5_ can easily accumulate in lung tissue and continuously stimulate the TGF-β pathway, causing damage to lung tissue [41]. Therefore, to evaluate the protective effect of the aqueous extract of *Codium fragile*, the pulmonary fibrous protein expression levels were confirmed. The administration of *Codium fragile* significantly attenuated pulmonary fibrosis (Figure 7). Tamarixetin, a quercetin derivative of *Codium fragile*, inhibited cardiac fibrosis by regulating the protein expression of TGF-β1, collagen I, collagen III, and matrix metalloproteinase-9 [42]. The consumption of gallic acid regulated hepatic fibrosis by regulating the mRNA levels of MMP-2 and tissue inhibitor of MMP-1 in carbon tetrachloride-induced liver injury mice [43]. In addition, morin as a flavonoid ameliorated allergic airway fibrosis by regulating the expression of matrix metalloproteinase-9 and cytokine levels of IgE and Th2 in bronchoalveolar lavage fluid in ovalbumin-induced mice [44]. Baicalein, one of the flavones in *Codium fragile*, inhibited pulmonary fibrosis by reducing microRNA-21 levels, which play an important role in the pathogenesis of pulmonary fibrosis, and by suppressing the up-regulated expression levels of TGF-β1 and p-Smad-2/3 in bleomycin-treated rats [45]. Based on these results, the aqueous extract of *Codium fragile* with phenolic compounds significantly suppressed PM_2.5_-induced lung fibrosis via the TGF-β1/matrix metalloproteinase/Smad pathway. Furthermore, it has been reported that lung fibrosis is sensitively affected by lipid changes [46]. Impairments and changes in fatty acid metabolism are associated with the pathogenesis of pulmonary fibrosis, and changes in the profile and metabolome of fatty acids are associated with disease progression and outcome [47]. The accumulation of triglyceride in the form of lipid droplets in alveolar epithelial cells induces endoplasmic reticulum (ER) stress and induces apoptosis of these cells through the expression of TGF-β1 [48]. On the other hand, stearic acid inhibited liver fibrosis by reducing α-SMA, collagen I expression, and ROS production in TGF-β1-induced fibroblasts [49]. In particular, ω-3 fatty acids have been reported to have excellent activity to improve pulmonary fibrosis [46]. In conclusion, *Codium fragile*, containing a large amount of unsaturated fatty acids and stearic acid, is judged to have an activity to improve pulmonary fibrosis by regulating the TGF-β1 pathway and changes in the profile and metabolome of fatty acids.

## 5. Conclusions

**Figure 8:**
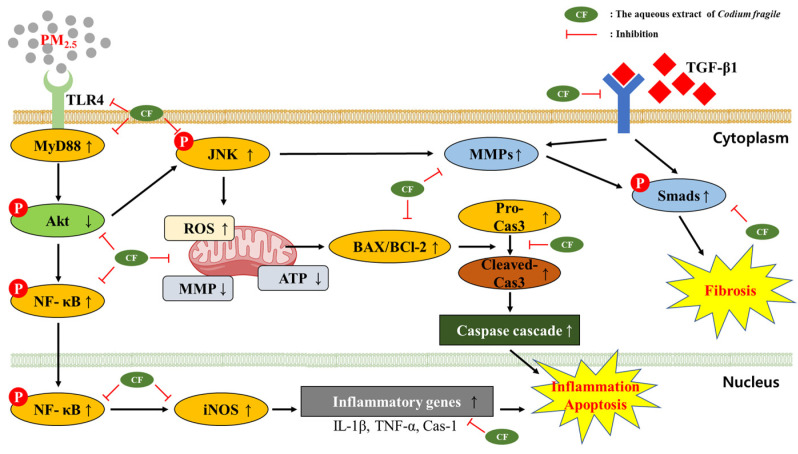
A schematic illustration presents the ameliorating effect of the aqueous extract of *Codium fragile* against particulate matter (PM)_2.5_-exposed pulmonary damage and fibrosis via TLR/TGF-β1 and NF-κB pathways. (↑) upregularation; (↓) downregulateion in image.

In conclusion, exposure to PM_2.5_ damaged the pulmonary antioxidant system and mitochondrial function and caused an inflammatory response, apoptosis, and fibrosis. However, the aqueous extract of *Codium fragile* had a protective effect against PM_2.5_-induced pulmonary cytotoxicity by regulating the TLR/TGF-β1/NF-κB pathway in BALB/c mice. This study proved that *Codium fragile*, a marine green alga, has a considerable and significant therapeutic effect against PM_2.5_-induced pulmonary damage, and might be a potential and beneficial resource for functional food to improve lung health (Figure 8). However, as discussed above, PM_2.5_ contains unspecific compounds such as heavy metals, VOCs, PAHs, and other organic compounds. Since the individual effect of these toxic materials is not clear, additional studies are needed. Moreover, additional studies related to genetic changes, nutritional studies, epigenetics, and effects on physiological activity of extracts of *Codium fragile* from exposure to PM_2.5_ due to environmental factors should be conducted.

## Figures and Tables

**Table 1:** List of antibody information.

Antibody	Catalog	Concentration	Manufacturer
TLR4	sc-52962	1:1000	Santa Cruz Biotech (Dallas, TX, USA)
MyD88	sc-74532	1:1000	Santa Cruz Biotech (Dallas, TX, USA)
p-JNK	sc-6254	1:1000	Santa Cruz Biotech (Dallas, TX, USA)
p-NF-κB	3033	1:1000	Cell Signaling Tech (Danvers, MA, USA)
iNOS	sc-7271	1:1000	Santa Cruz Biotech (Dallas, TX, USA)
Caspase-1	sc-392736	1:1000	Santa Cruz Biotech (Dallas, TX, USA)
TNF-α	sc-393887	1:1000	Santa Cruz Biotech (Dallas, TX, USA)
IL-1β	sc-4592	1:1000	Santa Cruz Biotech (Dallas, TX, USA)
BCl-2	sc-509	1:1000	Santa Cruz Biotech (Dallas, TX, USA)
BAX	sc-7480	1:1000	Santa Cruz Biotech (Dallas, TX, USA)
Caspase-3	CSB-PA05689A0Rb	1:1000	Cusabio (Hubei, China)
TFG-β1	sc-130348	1:1000	Santa Cruz Biotech (Dallas, TX, USA)
p-Smad-2	3108	1:1000	Cell Signaling Tech (Danvers, MA, USA)
p-Smad-3	sc-517575	1:1000	Santa Cruz Biotech (Dallas, TX, USA)
MMP-1	sc-21731	1:1000	Santa Cruz Biotech (Dallas, TX, USA)
MMP-2	sc-13595	1:1000	Santa Cruz Biotech (Dallas, TX, USA)
β-actin	66009-1-Ig	1:1000	Proteintech (Rosemont, IL, USA)

**Table 2:** Identification of main compounds of the aqueous extract of *Codium fragile* using GC/MS^2^ chromatography.

No.	RT (min) ^1^	Parent Ion	Fragment (*m/z*)	Compound
1	43.92	328	313, 269, 201, 117, 73, 43	Palmitic acid
2	48.58	356	341, 309, 241, 201, 117	Stearic acid
3	51.82	330	282, 249, 167, 149, 122	Oleamide

^1^ RT: retention time.

**Table 3:** Identification of main compounds of the aqueous extract of *Codium fragile* using UPLC-Q–TOF/MS^E^ chromatography.

No.	RT (min) ^1^	*m/z* [M + H]^+^	Fragment (*m/z*)	Compound
1	8.35	256	80, 88, 184, 201	Hexadecanamide
2	8.44	282	135, 149, 247, 265	Oleamide
3	8.73	338	80, 106, 309	13-docosenamide

^1^ RT: retention time.

## Data Availability

The data underlying this article are shared upon reasonable request to the corresponding author.

## Abbreviations

The following abbreviations are used in this manuscript.

BAX	BCl-2 associated X
BCl-2	B-cell lymphoma 2
GSH	Glutathione
IL-1β	interleukin-1β
iNOS	inducible nitric oxide synthase
MDA	Malondialdehyde
MMP-1	matrix metalloproteinase-1
MMP-2	matrix metalloproteinase-2
MMP	mitochondrial membrane potential
MyD88	myeloid differentiation primary response 88
NF-κB	nuclear factor kappa-light-chain-enhancer of the activated B cell
p-JNK	phosphorylated c-Jun N-terminal kinase
p-Smad	phosphorylated small mothers against decapentaplegic
PM	particulate matter
PM_2.5_	particulate matter, which classified as a size smaller than 2.5 μm
ROS	reactive oxygen species
SOD	superoxide dismutase
TGF-β1	transforming growth factor-β1
TLR	Toll-like receptors
TNF-α	tumor necrosis factor-α
